# Coping strategies in parents of children with chronic Adrenal insufficiency

**DOI:** 10.1192/j.eurpsy.2022.2276

**Published:** 2022-09-01

**Authors:** N. Faouel, R. Ben Soussia, K. Messai, M. Kacem, W. Bouali, A. Haj Mohamed, L. Zarrouk

**Affiliations:** 1 hospital Tahar sfar Mahdia, Department Of Psychiatry Mahdia, Mahdia, Tunisia; 2 Hospital Tahar sfar Mahdia, Department Of Psychiatry Mahdia, Mahdia, Tunisia

**Keywords:** parents, coping strategies, adrenal insufficiency, Children

## Abstract

**Introduction:**

Being the parent of a child followed for a chronic pathology can require different resources and coping skills.

**Objectives:**

to determine the adaptation strategies of the parents of children monitored for adrenal insufficiency in the face of their children’s pathology

**Methods:**

We conducted a descriptive cross-sectional study carried out with parents of children with Adrenal Insufficiency followed at the pediatric outpatient clinic in Taher Sfar Mahdia University Hospital between February 2019 and April 2020. We used a pre-established questionnaire collecting sociodemographic data and the strategies of coping using the Brief-COPE Board.

**Results:**

A total of 38 parents of children with adrenal insufficiency and 38 control parents participated in the study. The Brief-Cope board’s study of Coping strategies revealed that the strategies most used by parents of children with Adrenal insufficiency were, in descending order: religion (92.1%), support emotional (73.7%), distraction (63.9%), behavioral disengagement and acceptance (57.9%), instrumental support (52.6%), expression of feelings (50%), positive reinterpretation (39.5%), blame (38.9%), denial and humor (36.8%), active coping and planning (36.1%). On the other hand, those used by the control population were in descending order: religion (94.4%), distraction (84.2%), blame (78.9%), acceptance (72.2%) %), emotional support (69.4%), humor (63.9%), behavioral disengagement (61.1%), active coping (47.2%), expression of feelings (44 , 7%), planning (41.7%), instrumental support (30.6%), positive reinterpretation (22%), denial (19.4%).

**Conclusions:**

Psychological support for the parents of children with chronic illnesses would be necessary to prevent parental burnout and improve their ability to adapt to their experiences

**Disclosure:**

No significant relationships.

